# Predictable fibroblast tension generation by measuring compaction of anchored collagen matrices using microscopy and optical coherence tomography

**DOI:** 10.1080/19336918.2019.1644855

**Published:** 2019-07-22

**Authors:** Melville B. Vaughan, Gang Xu, Tracy L. Morris, Pratiksha Kshetri, Jing X. Herwig

**Affiliations:** a Department of Biology, University of Central Oklahoma, Edmond, OK, USA; b Center for Interdisciplinary Biomedical Education and Research (CIBER), University of Central Oklahoma, 100 N. University Drive, Edmond, OK; c Department of Engineering and Physics, University of Central Oklahoma, Edmond, OK, USA; d Department of Mathematics and Statistics, University of Central Oklahoma, Edmond, OK, USA

**Keywords:** Dupuytren’s disease, Optical Coherence Tomography, myofibroblast, tension generation, stress relaxation, 3D model, collagen lattice

## Abstract

The anchored fibroblast-populated collagen matrix (aFPCM) is an appropriate model to study fibrocontractive disease mechanisms. Our goal was to determine if aFPCM height reduction (compaction) during development is sufficient to predict tension generation. Compaction was quantified daily by both traditional light microscopy and an optical coherence tomography (OCT) system. Contraction in aFPCM was revealed by releasing them from anchorage. We found that aFPCM contraction increase was correlated to the compaction increase. Cytochalasin D treatment reversibly inhibited compaction. Therefore, we demonstrated that aFPCM height reduction efficiently measures compaction, contraction, and relative maturity of the collagen matrix during development or treatment. In addition, we showed that OCT is suitable for effectively imaging the cross-sectional morphology of the aFPCM in culture. This study will pave the way for more efficient studies on the mechanisms of (and treatments that target) migration and contraction in wound healing and Dupuytren’s contracture in a tissue environment.

## Introduction

Wound healing, Dupuytren’s contracture, and burn scars have in common connective tissue remodeling and contraction by fibroblasts, leading to the myofibroblast phenotype presence [1–3]. Fibroblasts *in vivo* use migratory properties to reorganize the collagen and generate sufficient tension so that myofibroblasts can differentiate [4]. The anchored, fibroblast-populated collagen matrix (aFPCM) or lattice model is an appropriate *in vitro* model to study these processes because migration/contraction mechanisms are well-known components [5,6]. Additionally, sufficient tension is generated to allow proliferation [7], an important factor in these processes, which as a group are often called fibroproliferative [8] or fibrocontractive [9]. Anchored collagen matrices form a low spherical-like cap with an apex; early articles [10,11] measured the reduction of this apex (i.e. compaction of the matrix) as a measurement of cellular activity. Once sufficient tension was generated, the matrix could be released from anchorage and allowed to recoil under contraction [12]; this was also used as a measurement of cellular activity [5,13,14]. Releasing the matrix has been a good method to indirectly measure the tension generated; contraction of the matrix is proportionate to the number of cells in the matrix, and the percentage of myofibroblasts [5,15,16]. Both compaction and contraction are processes involving fibroblast growth factor response, actin/myosin interactions, serum agonists, and are dependent on the number of cells and concentration of the collagen [17,18]. Using the anchored collagen matrix as previously published, compaction is required for contraction. Tension generation increases over time; there is an expected correlation between the compaction of the anchored matrix and its contraction shown through release; however, there are no known publications that address this correlation.

One common issue with wound healing and Dupuytren’s contracture is that migratory properties of fibroblasts reorganize connective tissue, leading to increased tissue tension [4,9]. This provides the passive biomechanical environment for signaling the myofibroblast phenotype [9]. Understanding these processes can have the potential to promote appropriate wound healing or to reduce the onset of overaggressive contractures. The anchored collagen matrix model has been useful to study the myofibroblast phenotype [6]. Fibroblasts in the collagen matrix use migratory mechanisms to remodel and compact the collagen, allowing tissue tension to increase [9]. Once sufficient tension is present, fibroblasts can be induced to form myofibroblasts, identifiable by the presence of the alpha-smooth muscle actin isoform assembled into cytoskeletal stress fibers [19,20]. Because of the clarity of the collagen matrix, whole-mount tissue (rather than thin sections) can be stained to identify proliferation [21], cytoskeletal elements, and extracellular matrix components [22] so that structure can be directly correlated to function.

Technically, it is not an easy task to measure the apex height of the collagen matrix. Early articles used microscope focusing through the depth of the collagen matrix to demonstrate compaction [10,11]. Follow-up studies showed that the matrix height reduction was mostly irreversible upon removal of contractile agonists or disruption of actin microfilaments [11]. Thus, the matrix height serves as a good measure for estimating increasing tension generation under control conditions. Using microscope focus to measure compaction (height reduction) requires fine resolution to identify collagen fibers at the apex of the matrix; additionally, it is difficult to identify the apex that is not always in the very center of the matrix.

Optical coherence tomography (OCT) was recently shown to effectively measure the cross-sectional contours of soft tissues including collagen matrices [23–25]. OCT employs low-power near-infrared light to non-invasively penetrate (up to 1–2 mm deep) into the scattering medium such as the thin translucent collagen matrices, and based on low-coherence interferometry with backscattered light, provides sub-surface cross-sectional imaging of the sample with micrometer resolution [26]. Therefore, compared with the regular microscope, OCT is a more convenient imaging tool to characterize the morphology of the aFPCM.

In this study, we first measured the height reduction during the maturation of the collagen matrix using both the microscope focus method and OCT. We showed that the microscope focus method provides an accurate measurement of the matrix apex height when compared with the OCT results. Secondly, utilizing the cross-sectional contour and area of the aFPCM from the OCT, we further estimated the volume of the collagen matrices by swiveling the relatively uniform cross-sectional area around its symmetric axis. The compaction of the collagen matrices during maturation, thus, was characterized by the significant reduction in not only the apex height, but also the matrix volume (while maintaining approximately constant anchorage area). Finally, we correlated the matrix compaction with the contraction through daily morphology measurements followed by matrix release measurements. This study will pave the way for other investigators to use the apex height reduction as an effective measure for compaction, tension generation, and relative maturity of the collagen matrix during development or treatment, leading to a more efficient study of migration and contraction mechanisms in wound healing and Dupuytren’s contracture.

## Materials and methods

### Cell culture, matrix preparation, and continued culture

All studies were approved by the Institutional Review Board of the University of Central Oklahoma. Briefly, normal human dermal fibroblasts (HDF01035) purchased from Lifeline Cell Technology (Frederick, MD 21,701), or Dupuytren’s Contracture fibroblasts (gift from James J. Tomasek, OUHSC) were grown in log-phase culture using DMEM/high glucose, 5% fetal bovine serum, and 1% antibiotic/antimycotic, in a 37°C, 5% CO_2_ incubator. Collagen matrices were set up and cultured as previously described [5,16] (0.65mg/ml rat tail type I collagen, 125,000 cells/ml) except the volume of matrix plated was reduced to 150 µl so that the entire matrix would fit in the imaging window of the camera used for matrix release. Matrices were cultured continuously at 37°C, 5% CO_2_, for up to 8 days, with ½ the volume of media replaced daily. Cytochalasin D (3 µM and 6 µM) was used as previously described [11,16] to study compaction inhibition.

### AFPCM apex height measurement with a light microscope

**Figure 1.**
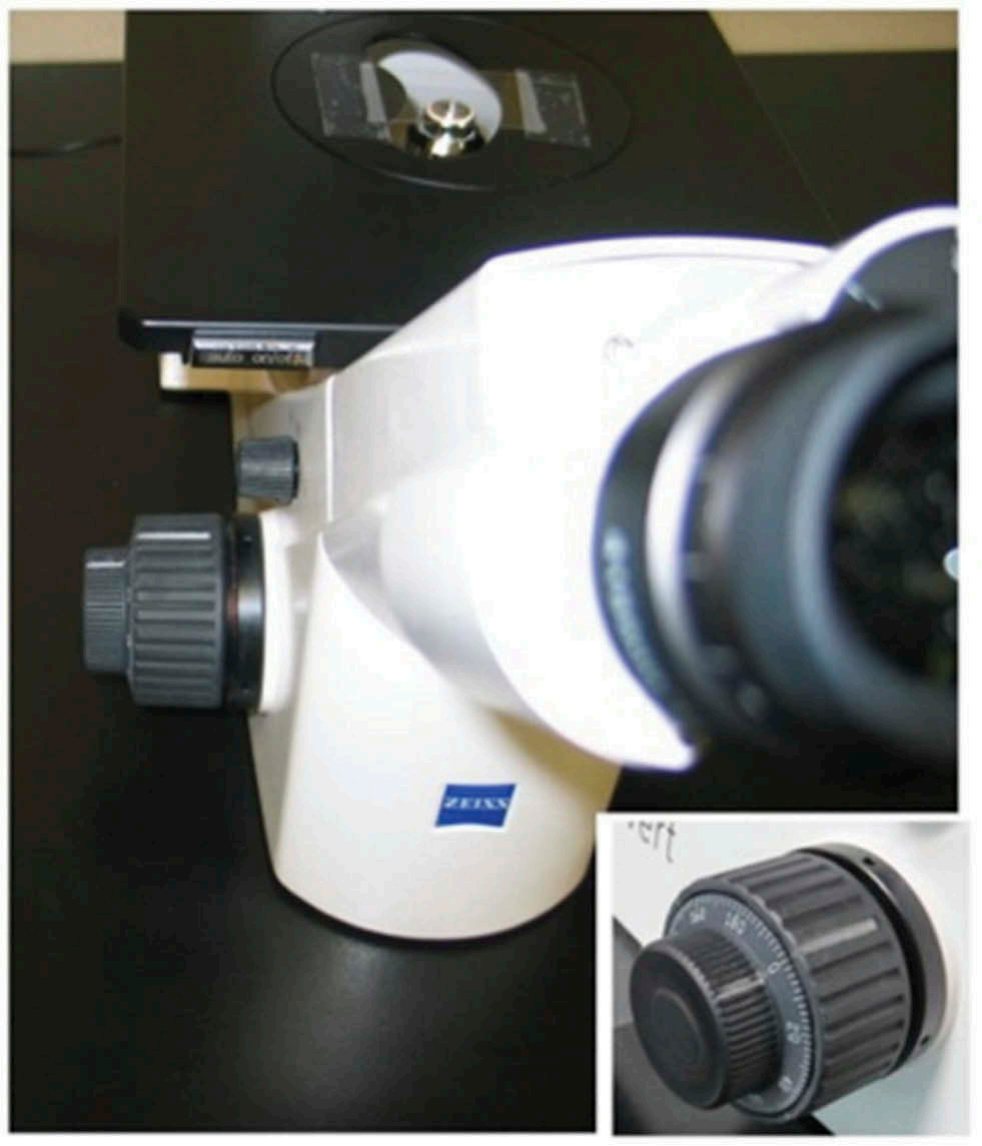
Setup for the matrix height measurement. A line was marked on both the fine and coarse focus knobs to facilitate unit measurements. Note the clockwise sequence of numbers on the fine focus knob (inset) requiring downward focusing movement to measure increasing distances.

An inverted light microscope (Zeiss Primovert) was used to measure the depth of the aFPCM using the 20x objective and the corresponding phase slider (Figure 1). Measurements were made using the number dial on the fine focus knob and a revolution marker placed on the coarse focus knob (Figure 1, inset). The measurements were calibrated by focusing through a microscope slide whose thickness was measured using a digital caliper. This calibration was 640 units per 1000 µm.

**Figure 2.**
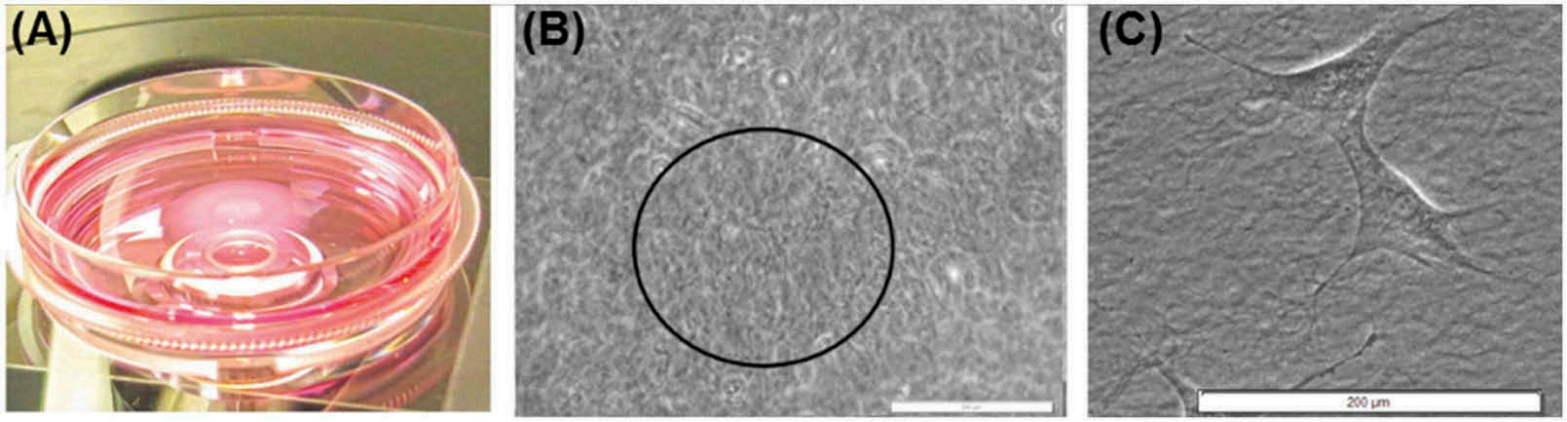
Apex height measurement of the aFPCM using the inverted light microscope. (A) The phase slider used for the 20x objective placed a small circle of light atop the aFPCM. (B) The apex of the matrix could be determined by the presence of a central region of focused collagen fibers (indicated in the black circle) surrounded by nonfocused fibers. (C) The bottom of the aFPCM could be determined by the presence of flattened cells on the substrate. The aFPCM apex height was then determined based on the number of revolutions of the fine focus knob between these two positions in (B) and (C). Scale bars in (B) and (C) represent 200 µm.

Each aFPCM was centered over the objective and under the circle of light (Figure 2A). The collagen fibers at the apex of the matrix were used to zero the fine focus wheel (Figure 2B). The aperture lever on the condenser diaphragm was closed sufficiently to increase the collagen fiber resolution as necessary. Depth measurement was complete when the flattened cells on the plate beneath the aFPCM came into focus (Figure 2C). Focus wheel measurements were collected and transformed into micrometers using the above calibration.

### Matrix morphology measured by OCT

**Figure 3.**
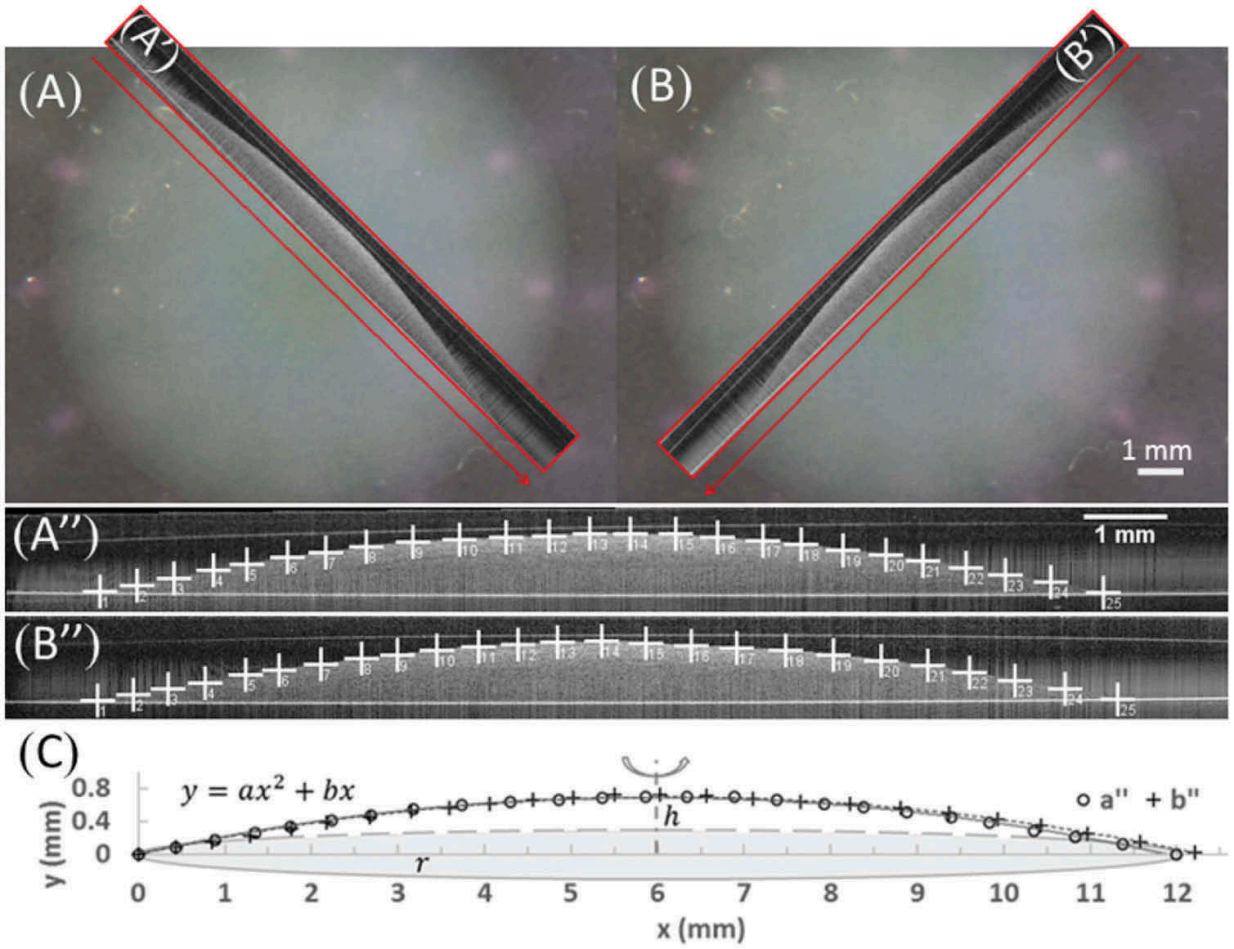
Matrix morphology measured by OCT. (A, B) video graphs of the same collagen matrix. The red line indicates the location for 2D cross-sectional view by OCT. (A’, B’) The 2D cross-sectional side view images of the sample from OCT. (A’’, B’’) Manual tracing the dome contour of the collagen matrix from OCT. (C) Fitting the contour with a polynomial function and swiveled into a 3D dome.

In order to confirm the collagen matrix height measurements from the microscope, we further characterized the matrix morphology by imaging the cross-sectional shape of the collagen matrix with an OCT imaging system (Thorlabs, Newton, NJ) as previously described [26]. The low-power near-infrared light can noninvasively penetrate into the scattering sample of the 1- to 2-mm thin translucent collagen matrix, and based on low-coherence interferometry with backscattered light, provide a clear visualization of the 2D cross-sectional contour and area of the matrix in two diagonal directions (Figure 3). Immediately after OCT imaging, aFPCMs were returned to the incubator and subsequently imaged each following day. Based on the contour, the apex height (h), the base radius (r) and area ($A=\pi r^{2}$) were readily determined. In addition, the cross-sectional contour of the matrix was manually traced by inserting 20–30 points in ImageJ (Figure 3(A’’,B’’)), and their (x, y) coordinates were fitted with a quadratic polynomial function (Figure 3C) as

(1) $$y=ax^{2}+bx$$

where a and b are constants. For each matrix imaged on a particular day, there were two sets of characterized contours from different diagonal directions that were well matched (e.g. the two shown in Figure 3). Assuming the axis symmetry of the matrix (as confirmed by almost identical contours in two diagonal directions; see Figure 3(A’’,B’’), the volume of the dome-like collagen matrix was estimated by swiveling the contour around the symmetric axis (Figure 3C) as

(2) $$V=\int_{0}^{r}y\cdot 2\pi \left(r-x\right)dx$$

Inserting Equation 1 into 2, we completed the integration to obtain

(3) $$V=\frac{\pi }{6}ar^{4}+\frac{\pi }{3}br^{3}$$

Thus, equation 3 provides a convenient formula to estimate the volume of the dome-like collagen matrix. Note that if the apex height and the base radius were determined entirely based on the quadratic polynomial function (Equation 1) as the maximum y function value at the specific *x* location, Equation 3 could be further simplified as

(4) $$V=\frac{1}{2}\pi r^{2}h$$

We used Equation 3 to calculate the volume of the dome-like collagen matrices during development and showed in Discussion that Equation 4 would produce nearly identical results.

### Matrix release to reveal mechanical tension

**Figure 4.**
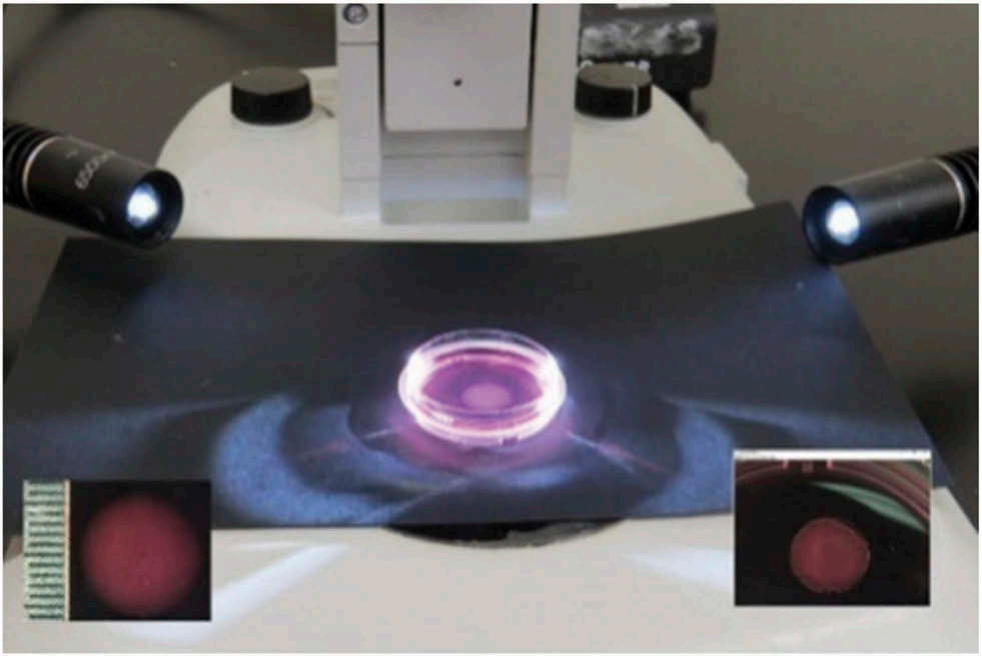
Matrix-release experiment. Placement of the light source lateral to the matrix dishes reduced reflected glare from the subsurface. The left inset shows a photograph taken of an attached matrix including a measurement ruler used to calibrate the ImageJ software measurement program. Each tick equals 1 mm. The right inset is an ImageJ software window showing the released aFPCM with yellow circle used by the program to calculate the matrix area.

Every day following aFPCM height measurements, 3 dishes with matrices were removed from further height measurement and used for matrix-release measurements. Matrix measurements were done using photographs rather than using a ruler to measure diameters as previously reported [5,16]. The matrix was placed atop a black surface directly under a stereo dissecting microscope lens (Olympus SZ61), while two fiber optic light sources (AmScope) were placed lateral to the dish to highlight the aFPCM while reducing the background glare (Figure 4). Each matrix was photographed before release from its attachment to the substrate (time 0) using a digital camera (SPOT diagnostics) (Figure 4, left inset). The edge of the matrix was then lifted with a probe or spatula, then using a disposable plastic pipette about 500 µl of a 1 ml volume was quickly and deliberately flushed under the lifted edge to release the matrix from the dish. Matrices were returned to the incubator and photographed at 1, 2, 10, 30, and 60 min after release. The degree of matrix area reduction after removal from anchorage served as an indicator of the magnitude of the contractile stress or tension in the collagen matrix. The larger the mechanical tension in the matrix relative to its elasticity [27], the smaller the matrix area would be pulled into right after release. Area reduction of matrices was analyzed using ImageJ software (1.46r; NIH). The edge of each matrix was encircled (Figure 4, right inset) and measurement reported in millimeters squared after calibrating the images to 87 pixels per millimeter using an image taken of a ruler at the same magnification (Figure 4, left inset).

### Statistical analysis

A 3-factor analysis of variance (ANOVA) was performed, with fixed factors for experiment and treatment (levels: 3um cyto-d, 6um cyto-d, and control), and a repeated factor for time (levels: 0, 1, 2, 3, 3.05, 4, 5, 6, 7, 8, 9, 10, 11, and 12 days). The response variable, height, was log-transformed to normalize the residuals. A heterogeneous autoregressive lag 1 structure was used to model the correlations between successive measurements over time. The ANOVA was followed by Tukey’s multiple comparisons when necessary. All statistical analysis was performed in SAS v. 9.4 using proc mixed.

## Results

### Matrix compaction increases over time

**Figure 5.**
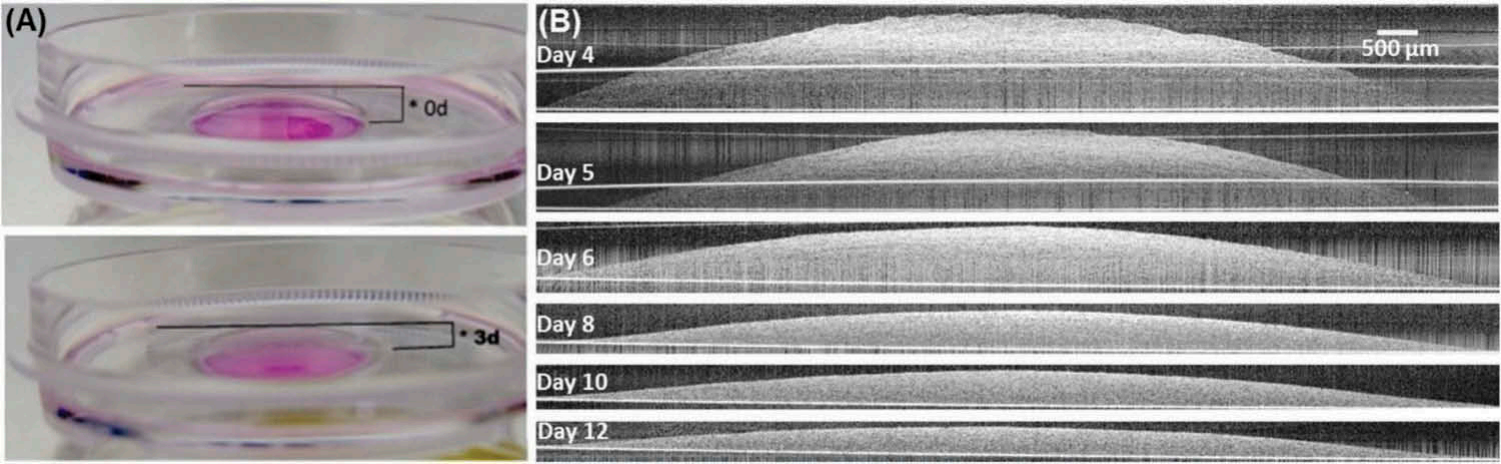
Matrix compaction increases over time. (A) Matrix height after 3 days of culture was visibly reduced (bottom) compared to the initial day (top). Media was removed to enable view. (B) Cross-sectional side view of the developing collagen matrix from day 4 to 12 under OCT. The matrix appeared as a spherical cap-like shape with an apex at its center and thin attachment at the periphery. The matrix height visibly decreased during development while the anchorage area remained about constant.

**Figure 6.**
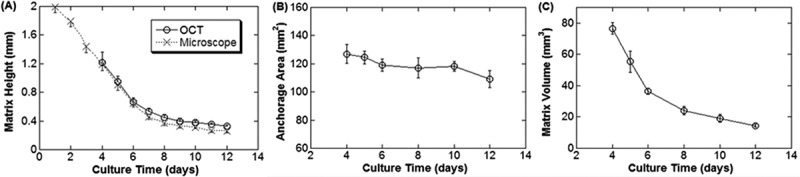
Matrix morphology measurement with OCT and light microscope. (A) The matrix height during development measured with both microscope and OCT. Note the close agreement of the data from these two methods. (B, C) The anchorage area and volume of the same set of collagen matrix from day 4 to day 12 measured with OCT.

The matrix compaction was characterized by two parameters: apex height and matrix volume. The matrix height was visibly reduced after 2 to 3 days of culture while maintaining similar anchorage area (Figure 5A). For one of the culture sets, the apex height was measured by the microscope focus method to be about 2 mm at the beginning of culture and rapidly decreased to less than 1 mm on day 5 (Figure 6A). Afterward, the matrix compaction continued and reached the maximum as the height reached its minimum of about 0.3–0.4 mm after day 7 (Figure 6A). As the matrix approached maximum compaction it became challenging to identify the surface under the light microscope due to interference from the crowded matrix.

Morphology of the developing collagen matrix was further characterized by OCT. For the same set of culture, from day 4 when the matrix height fell within the OCT imaging depth of about 1.5 mm, the cross-sectional side view of the collagen matrix was readily visualized to be a rounded dome with an apex (Figure 5B). Both the height and cross-sectional area of the matrix was progressively reduced during the subsequent culture days (Figure 5B). Specifically, the apex height measurements by OCT from day 4 to 12 agreed well with those by the microscope focus method (Figure 6A). During this height reduction, the anchorage area of the matrix remained more or less constant (Figure 6B). As a result, the volume of the matrix, as determined by swiveling the cross-sectional area about the center axis, decreased considerably from about 75 mm^3^ on day 4 to about 15 mm^3^ on day 12 (Figure 6C).

### Cell activity is required for matrix compaction

**Figure 7.**
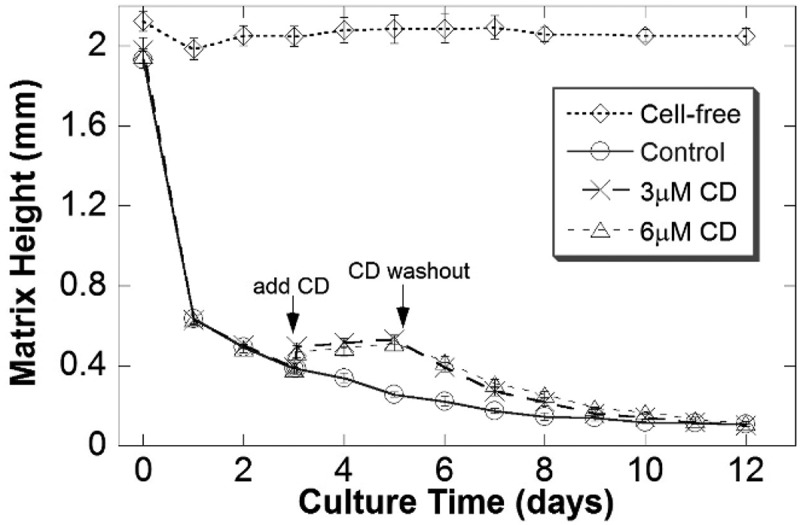
Compaction requires cell presence and an intact actin cytoskeleton. Matrices with cells compacted with reduced heights, while cell-free lattices did not. Treatment with the cytoskeleton disruptor cytochalasin D (CD; either 3 µM or 6 µM) on day 3 relaxed the matrix to the larger height in just 1 h; washout of CD with growth media on day 5 allowed compaction to continue from day 6 and resume to the same level as the control on day 9.

The collagen matrix compaction observed in Figures 5 and 6 required the presence of fibroblasts, as cell-free collagen matrices did not compact at all during development (Figure 7). Furthermore, disruption of the actin cytoskeleton in the cells using cytochalasin D (CD) in the middle of development (day 3) slightly relaxed the matrix as indicated by increased height from after one-day (day 4) of CD treatment (Figure 7). There was little difference between the 3 µM and 6 µM doses of CD. This relaxation effect was reversible upon washout of CD on day 6, as indicated by resumed height reduction and matrix compaction after removal of CD (Figure 7). The three-way interaction between experiment, treatment, and time was significant (F = 3.37, p < 0.0001); There was a significant difference in the treatment means at times 3.05–10 days. For times 3.05–8 days, the control mean was significantly lower than both of the CD treatment means (p < 0.0001), but there was no significant difference between the two CD treatment means at any of these times. For times 9–10 days, the control mean was significantly lower than the 6 µM CD treatment mean (p < 0.0001), but there were no other significant differences.

### Matrix contraction increases over time

**Figure 8.**
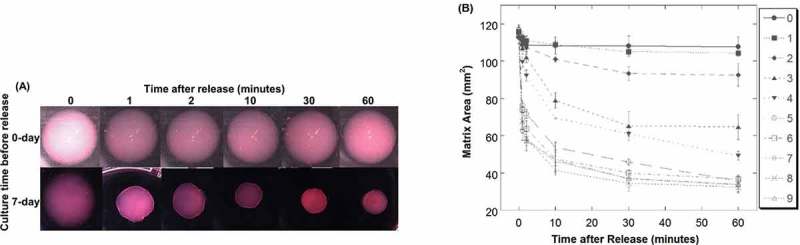
Reduction of matrix area after release increases over time. (A) Video graphs of released collagen matrices from day-0 and day-7 culture. B) Matrix area reduction after release from Day 0 to 9 cultures. Numbers in the legend indicate days in culture before release.

Matrices released from anchorage immediately after polymerization reduced the area very little over time (Figure 8A, top; Figure 8B-Day 0), suggesting little or no contraction from cells or mechanical tension on the collagen matrix. Over the next few days, the matrix area decreased more rapidly and to a greater extent upon release, suggesting increased cellular contraction or mechanical tension on the matrix over time (Figure 8B). The area reduction after release reached a maximum after about 7 days (Figure 8A, bottom; Figure 8B-Day 7). Note that the matrix area reduction after release showed a biphasic contraction: an initial rapid recoil that occurred within the first 10 min, followed by a slow further reduction. The time rate and magnitude of area reduction for both phases changed during developing days: the rapid recoil became faster and further, while the ensuing slow reduction approached zero.

### Increased matrix contraction is correlated with increased compaction

**Figure 9.**
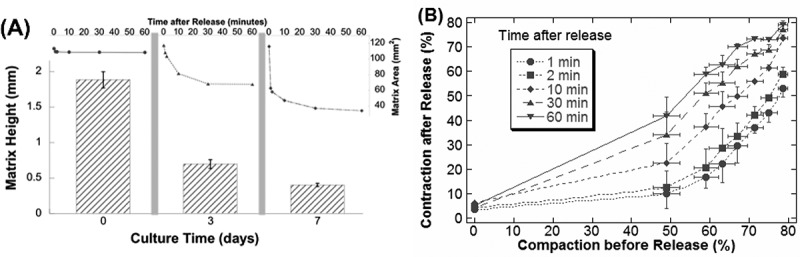
Correlation between matrix contraction and compaction. (A) Isolated culture-time graphs demonstrate the predictable increase in tension that occurs with increased compaction. Note in the line graphs, the increase in contraction within the first 10 min after release, suggesting that tension increases with culture time. (B) Compaction is defined as the percent reduction of the matrix height before release, and contraction is defined as the percent reduction of the matrix area after release. Data points shown are mean and standard deviation (only one direction shown for contraction for clarity).

So far, we have separately observed and measured the matrix compaction (as indicated by matrix height or volume reduction) and tension generation (as indicated by matrix contraction after release from anchorage) during development. It is intuitive to expect that both processes are correlated to each other. When at each extreme, the compaction and contraction amount were correlated: initially at day 0, there was neither compaction nor contraction; by about day 7, compaction reached a maximum as did contraction (Figure 9A, data extracted from Figures 6 and 8). Therefore, if we define compaction as the percent reduction of the matrix height before release and contraction as the percent reduction of the matrix area after release, matrix contraction could be plotted against the compaction directly. Clearly, there is a positive correlation between contraction and compaction: the more compaction the matrix has had before release, the more contraction the matrix would exhibit after release (Figure 9B). The linearity of this correlation became more apparent for matrix compaction that was larger than about 50%, which was only after one-day culture (Figure 9B).

## Discussion

The anchored collagen matrix is one of the many models that is used to study fibroblast biology, and one of only a few models appropriate to study myofibroblast biology. Because fibroblasts are specialized mechanical and material cells of vertebrate soft tissues [28], this model is an appropriate fibroblast structure/function model. Recent studies provided evidence that the anchored collagen matrix compaction was comparable to wound healing granulation tissue formation, and that released matrix contraction was comparable to the scar formed at the end of wound healing [29]. Many investigators instead use the free-floating or compliant matrix (released from anchorage as soon as it has polymerized) to study tractional force generation [17] in part because it is easier to measure, but also because cells typically do not proliferate in a compliant matrix [7,30] which is an important variable affecting compaction [31]. Unfortunately one cannot use the compliant matrix to study myofibroblasts [6]. It is important to understand how much matrix reorganization is necessary to observe proliferation or the myofibroblast phenotype. In our unpublished immunostaining data, neither myofibroblasts nor proliferation were observed until 2 days of compaction had occurred. This suggests that experiments designed to reduce already-present myofibroblasts would not be relevant until after 2 days of compaction. Therefore, leaving the matrix anchored and measuring the height reduction over time as compaction would be very useful for investigators who want to study the myofibroblast phenotype using a 3D model in a more efficient way. Our results show that although it is difficult, the matrix compaction can be monitored over time using simple light microscopy for those who do not have OCT technology, because the apex height measurements match those collected using optical coherence tomography. A combination of regular light microscopic imaging with different suitable imaging techniques for the collagen matrix, such as OCT and multiphoton tomography [24], have the potential to increase the quality of the data collected for the morphology and structure of developing collagen matrix. The aFPCM provides sufficient structural support, material stiffness and collagen concentration [32] to sustain increased mechanical tension, allowing for both differentiation and proliferation. This is important for studying Dupuytren’s contracture where proliferation may be more important than migration for disease progression [33]. When the tension was released using cytochalasin D (disruption of stress fibers) the loss did not return the collagen matrix height to its initial size. This irreversible remodeling of the matrix was demonstrated previously [11] and mimics *in vivo* contracture. For this reason, the anchored collagen matrix is an appropriate model for studying contracture biology [28,34].

Although we showed that it can serve as a reasonable indicator for both compaction and tension generation of the anchored matrix, the height reduction does not result from cell contraction between top and bottom of the anchored matrix. From the mechanics point of view, the magnitude of the tension, if any, between the top and bottom of the anchored matrix should be much smaller, due to lack of support on the top, than that of the tension in the anchored plane (similar to thickness reduction of an elastic membrane while being stretched). Furthermore, as stated earlier, compaction of the compliant matrices is based on migratory mechanisms, not cell contraction due to lack of anchorage on the matrix apex.

The significance of this study lies in the correlation data between height reduction and delayed-release contraction (see Figure 9). With this data, an investigator may predict whether sufficient tension has been generated to study the myofibroblast phenotype. Different cells compact the matrix differently so it is crucial that the investigator monitors the compaction. Also, it is novel to compare contraction between different days of matrix release data. Note that there are two parts to the matrix contraction (Figures 8B and 9A), the fast contraction that happens in the first 2–10 min after release and the further slower contraction afterward. We previously showed that stress fibers have broken down within the first 10 min after release [16]. It can then be predicted that the early released-matrix contraction is mediated by mechanical tension in stress fibers, while the ensuing slower contraction is likely due to cell migration in the now-compliant matrix. Therefore, the amount of tension generated is correlated to the early fast contraction. Our data show this part of the graph to increase each day the matrix remains anchored, demonstrating a characteristic biphasic contraction that is consistent with data published elsewhere [5,12–16,35,36]. Theoretically, maximum tension generation would be demonstrated upon matrix detachment by an immediate and substantial diameter reduction (i.e. a steep initial slope), leaving little ability to contract afterward (i.e. little or no second slope). In summary, one can predict how much tension has been generated by the compaction measurement, and confirmed by viewing the anchored-release contraction graph.

The primary goal of this study was to show that matrix compaction was correlated to its tension generation. Previous studies have determined that matrix compaction is mostly, if not entirely, mediated by cell migration mechanisms [17,37], while matrix contraction immediately after release from the anchorage is mediated by stress fiber breakdown correlated with cell contraction [16,37]. Although not directly caused by stress fiber-mediated tension generation, our data showed that matrix height reduction or compaction can serve as a good indicator for the level of tension generated in the anchored matrix, which has been traditionally measured by matrix area contraction following release (sacrifice) from the anchorage.

Our data are consistent with previously published results [10,11] showing that anchored matrix compaction matures to a maximum level under control conditions, and can be altered during the maturation process. Compaction requires cellular activity including an intact actin cytoskeleton and is mediated by migration-like tractional force generation. Furthermore, we demonstrated a correlation between compaction and tension generation: the more compaction the matrix undergoes before release, the more contraction the matrix would exhibit after release, suggesting more tension generation. However, this correlation is best understood as a potential for tension generation or contraction under control conditions. When the tension generation is disrupted, the compaction does not relax to the same degree as the contraction and so the correlation is also disrupted. The cytochalasin D results were included to demonstrate our method of measuring matrix height would yield results similar to those already published [11,16], but additionally, we provided new data on the consequence of cytochalasin D washout that the cells would continue remodeling the matrix. This had been published previously, but only with free-floating (stress-relaxed or compliant) matrices [16]. Therefore, we provide good correlative data of the similarity of compaction to floating matrix contraction. This is not surprising as the compaction process involves reorganization of collagen fibers, not merely passive mechanical deformation of the network by cells [11]. When the compaction reaches a maximum, the stiffness and concentration of collagen would provide a tensile environment approaching that of the 2D models, albeit with cells completely surrounded by matrix providing a more in the vivo-like environment [38,39]. A compromise between the 3D anchored matrix and the stiff 2D environment was developed using collagen plated atop acrylamide gels of varying concentrations [40]. This model was essential to determine the amount of tension necessary for myofibroblast formation [41]. The aFPCM, monitored over time, should be able to provide similar information in a 3D environment.

Fibroblasts can sense the stiffness of the matrix and respond in a process termed tensional homeostasis [42]. The immediate contraction of the collagen matrix after release from anchorage suggests that considerable mechanical tension exists in the matrix before release. The existence of mechanical tension in the anchored collagen matrix could also be indicated by smoothly curved surface contours (see Figure 5B). If this developing mechanical tension is sufficiently large relative to the matrix stiffness during maturation, it would be expected to minimize the free surface area of the collagen matrix. To examine this speculation, we compared the volume of the estimated collagen matrix (Equation 3) with that of a spherical cap that possesses the same bottom radius, r and apex height, h (see Figure 3C for these two parameters). Both volumes were normalized by the volume of a circular cylinder with the same radius and height (thus, all ratios are expected to be less than 1). As the volume of the collagen matrix dome was estimated using Equation 3, the volume of the spherical cap and the cylinder can be, respectively, calculated as

(5) $$V_{sph}=\frac{1}{6}\pi h\left(3r^{2}+h^{2}\right)$$

and

(6) $$V_{cylinder}=\pi r^{2}h$$

**Figure 10.**
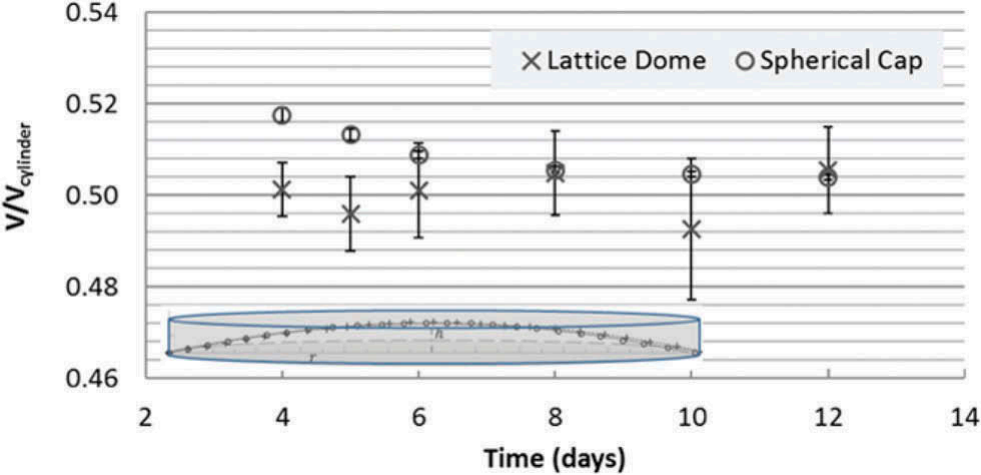
The shape and volume of the collagen matrices closely resemble those of spherical caps.

Note that if h≪r (as for most of the matrix dome during later maturation day; see Figure 5B), the volume of the equivalent spherical cap would be reduced to.

(7) $$V_{sph}\approx \frac{1}{6}\pi h\left(3r^{2}\right)=\frac{1}{2}\pi r^{2}h$$

which became identical to Equation 4. As a result, the volume ratio of the spherical cap over the cylinder (Equation 5 divided by Equation 6) would be expected to approach 0.5 using matrix data during compaction. Therefore, using 0.5 as the baseline, we plotted and compared the volumes of the collagen matrices and the equivalent spherical caps (both divided by the corresponding volumes of circular cylinders) during maturation (Figure 10). We found that the estimated volumes of the collagen matrix from our experiments are almost identical to those of equivalent spherical caps, with the maximum difference being about 5% (Figure 10), which suggests that the free surface area of the collagen matrices is approximately spherical resulting from mechanical tension developed within the matrix.

To summarize, we showed that the height reduction can serve as a simple indicator for both compaction and contraction (tension generation) of the aFPCM in control conditions. The direct measurement of compaction can be used to estimate the potential of contraction or tension generation in the collagen matrix, which would help improve the experimental efficiency (less or no need to sacrifice the anchored matrices to measure contraction). Furthermore, we showed that the anchor-release contraction measurement can serve as an indicator of tension maturity in the matrix. This will maximize the study of cell contraction and minimize cell migration when compaction reaches a maximum. In addition, we also showed that OCT can provide more information on the matrix morphology and make it easier to quantify the compaction. However, the shape measurements, including heights of anchored matrices and areas of released matrices, could only provide qualitative information about the relative mechanical tension generated in the matrix. Although the mechanical tension in other collagen matrix systems has been quantified or controlled through supporting posts or rings [27,43,44], little has been done to quantify and monitor the mechanical tension in the anchored collagen matrix [45]. For that to occur, one would need measure not only the area contraction (deformation) of released matrices, but also the mechanical properties (linear/nonlinear elasticity or viscoelasticity) of developing collagen matrices, as regulated by mechanics of any materials. Therefore, further work in this regard is warranted. Quantitative information on tension generation and its regulation are needed to help evaluate tissue remodeling and cell contraction during development and in response to wounds and fibrotic diseases. Technological advances including designer hydrogels [46], tunable collagen or aligned fibrils [47,48], self-assembled matrices [49] and bioprinting [50] will benefit from tissue behavior monitoring using OCT.
